# Stage-Specific Collagen V Knockdown Reveals Temporal Control of Fibrillogenesis, Remodeling, and Mechanics in Tendon Healing

**DOI:** 10.3390/ijms27104551

**Published:** 2026-05-19

**Authors:** Brittany L. Taylor, Ryan J. Leiphart, Michael S. DiStefano, Stephanie N. Weiss, Harrison B. Broadaway, Louis J. Soslowsky

**Affiliations:** 1 J. Crayton Pruitt Family Department of Biomedical Engineering, University of Florida, Gainesville, FL 32611, USA; brittany.taylor@ufl.edu (B.L.T.);; 2 McKay Orthopaedic Research Laboratory, University of Pennsylvania, Philadelphia, PA 19104-6081, USA

**Keywords:** tendon, collagen V, biomechanics, healing, extracellular matrix

## Abstract

Collagen V is a minor fibrillar collagen that regulates type I collagen fibrillogenesis; however, its time-dependent role during the stages of adult tendon healing remains unclear. We investigated the stage-specific effects of inducible *Col5a1* knockdown during tendon repair in a murine injury model. Collagen V expression was transiently suppressed during either the late inflammatory phase (tamoxifen-induced knockdown at 5 days post-injury; TM5) or early remodeling phase (tamoxifen-induced knockdown at 21 days post-injury; TM21), with outcomes assessed using gene expression, ultrastructural, and mechanical analyses. Early knockdown at 5 days post-injury, TM5, was associated with increased fibril diameter, greater fibril heterogeneity, and reduced structural mechanical properties, including decreased stiffness and maximum load. In contrast, delayed knockdown at 21 days post-injury, TM21, imposed after initial fibril organization, resulted in minimal changes in quasi-static mechanics but altered viscoelastic behavior and late-stage gene expression. An allele dose-dependent response was observed, with complete *Col5a1* knockdown producing greater structural disorganization and mechanical deficits. Transcriptional changes suggested time-dependent effects on extracellular matrix regulation, including proteoglycans, remodeling enzymes, and tenogenic markers. Collectively, these findings indicate that collagen V may function as a time- and dose-sensitive contributor to tendon healing, with a critical role during early fibril organization. Disruption during this time window has lasting effects on tendon structure and mechanics, informing stage-specific therapeutic strategies for tendon repair.

## 1. Introduction

Tendons are the primary connective tissue that link muscle to bone, transmitting the tensile forces generated by muscle contraction to the skeletal system and initiating locomotion. Their mechanical performance derives from a highly ordered extracellular matrix (ECM) that is organized at multiple structural levels, with fibers oriented parallel to the primary load axis. This tissue-specific organization is essential for the tendon’s capacity to sustain large, repetitive tensile loads while maintaining viscoelastic compliance. Collagen type I constitutes the majority of the tendon matrix and is the key determinant of tensile strength. In addition to providing structural integrity, the orientation and packing density of type I collagen fibers modulate the biomechanical properties of the tendon [1,2]. Although collagen I is the predominant ECM constituent and well recognized as central to tendon function, it is increasingly evident that less abundant collagens, such as collagen type V, play critical roles in establishing and maintaining matrix organization [3,4].

Collagen type V has emerged as a pivotal regulator of tendon biology. Despite being in relatively low abundance, collagen V is a critical driver of collagen I fibril assembly [3,4]. It co-assembles with collagen I during fibrillogenesis and protrudes from the fibril surface to constrain lateral growth and thereby determine fibril diameter and spacing [5]. These nanoscale parameters translate directly to the larger-scale mechanical properties of the tendon. Deficiency in collagen V is linked to disrupted fibril architecture and impaired mechanical integrity [6,7]. Recent studies have highlighted the importance of collagen V in development, healing, and pathological states such as Ehlers–Danlos syndrome [8,9,10,11,12], underscoring its role as a gatekeeper of matrix architecture.

In prior work, we demonstrated that murine collagen V haploinsufficiency results in significantly impaired recovery of tensile strength and aberrant collagen fibril morphology following acute tendon injury [11]. These studies utilized conventional knockout mice in which collagen V is inactivated from embryogenesis, providing valuable insight into the role of collagen V during injury responses within a collagen V-deficient tissue. However, this model does not allow investigation of the role of collagen V in the maintenance and injury response of mature, healthy tendon tissue; observed phenotypes may result from developmental abnormalities due to collagen V knockdown rather than time-dependent specific regulatory functions of collagen V during mature tendon repair. Therefore, the isolated contributions of collagen V at defined phases of adult tendon healing remain unknown.

To address this knowledge gap, we employ a tamoxifen-inducible Cre/loxP animal model that permits temporally precise ablation of *Col5a1* expression. This model provides the opportunity to elucidate the isolated role of collagen V during defined post-injury healing phases. Additionally, by generating both *Col5a1* null and heterozygous mice with temporal control, we can parse out the contributions of partial versus complete knockdown loss of *Col5a1* expression throughout repair. Recent work in which *Col5a1* expression was knocked down at the time of patellar tendon injury in an allele dose-dependent manner demonstrated that collagen V is essential for maintaining the mechanical integrity and homeostasis of mature, healthy tendon tissue [6]. Moreover, these findings revealed that collagen V plays a dynamic, dose-dependent role in tendon healing, such that modulation of its expression differentially influences post-injury structural organization and functional outcomes [6,7].

In this study, we investigate the mechanistic effects of partial versus complete *Col5a1* knockdown during the late inflammatory (4–7 days post-injury) and remodeling (7–21 days) phases of tendon healing. Our objective is to define the stage-specific roles of collagen V and determine how its downregulation during these critical windows modulates the reparative cascade. We hypothesize that altering *Col5a1* expression during the late inflammatory and early remodeling phases of healing will disrupt tendon repair, leading to a significant inflammatory response, impaired matrix organization, and inferior mechanical properties compared with wild-type injured controls. By integrating gene expression, ultrastructural, and biomechanical analyses, this study delineates how temporal modulation of *Col5a1* expression influences hierarchical matrix organization and the downstream consequences for tendon repair.

## 2. Results

### 2.1. Gene Expression

**Figure 1:**
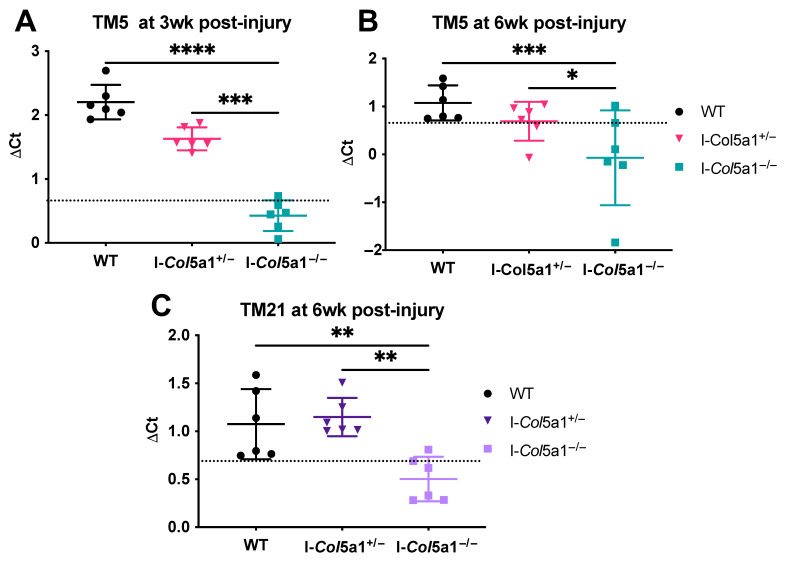
*Col5a1* expression is significantly reduced in heterozygous (I-*Col5a1*^+/−^) and null (I-*Col5a1*^+/−^) knockdown tendons relative to injured wild-type (WT) control tendons when knockdown was induced at 5 days post-injury (TM5) after (**A**) 3 weeks and (**B**) 6 weeks of healing, and at (**C**) 21 days post-injury after 6 weeks of healing. Allele dose-dependent reduction in *Col5a1* expression was also observed between the I-*Col5a1*^+/−^ and I-*Col5a1*^−/−^ tendons across induction and healing time points. Dotted lines denote *Col5a1* expression in uninjured WT tendons. Data is displayed as mean ± standard deviation with individual data points. One-way ANOVAs and Tukey post hoc corrections to identify pairwise differences. Significant comparisons are denoted by solid bars. (* *p* < 0.05, ** *p* < 0.01, *** *p* < 0.001, and **** *p* < 0.0001).

We first assessed the knockdown model’s efficacy by comparing *Col5a1* expression across genotypes at each induction and healing time point. Inducible knockdown tendons, I-*Col5a1*^−/−^, exhibited significantly reduced *Col5a1* expression compared to WT and I-*Col5a1*^+/−^ tendons at TM5 3 weeks (Figure 1A), TM5 6 weeks (Figure 1B), and TM21 6 weeks (Figure 1C). Together, these findings confirm that the knockdown models reflect the intended allele dose-dependent reduction in *Col5a1* expression. Gene expression analysis was also performed to assess the effects of *Col5a1* knockdown timing and allele dosage on matrix remodeling, fibrillogenesis, and tenogenic marker expression during tendon healing. A summary of the findings is outlined in Table 1.

#### 2.1.1. Matrix Remodeling and Proteolytic Enzymes

**Figure 2:**
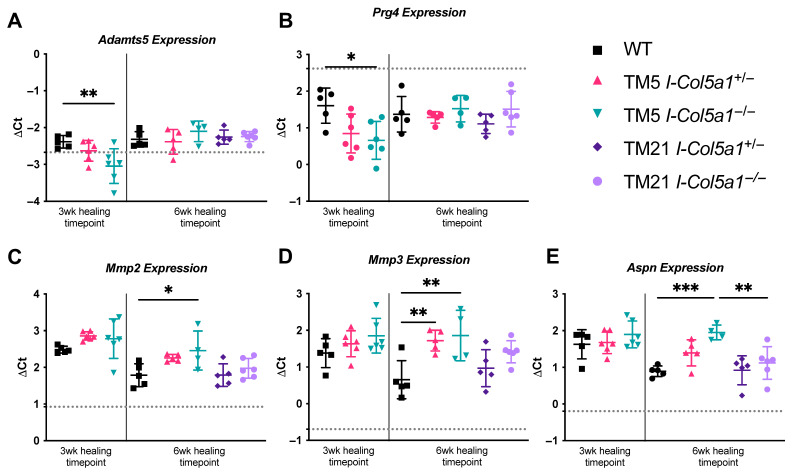
(**A**) *Adamts* and (**B**) *Prg4* expressions were reduced in tendons with *Col5a1*^−/−^ knockdown at 5 days post-injury (TM5 I-*Col5a1*^−/−^) relative to injured wild-type (WT) controls after 3 weeks of healing. After 6 weeks of healing, (**C**) *Mmp2*, (**D**) *Mmp3*, and (**E**) *Aspn* expressions in TM5_I-*Col5a1*^−/−^ tendons and (**D**) MMP3 expression in TM5_I-*Col5a1*^+/−^ tendons were upregulated relative to injured WT controls. Further, (**E**) *Aspn* expression was markedly reduced in null *Col5a1* knocked down at 21 days post-injury (TM21_I-*Col5a1*^−/−^) relative to its counterparts that were induced at 5 days post-injury (TM5_I-*Col5a1*^−/−^). Dotted lines denote gene expression in uninjured WT tendons. Data is displayed as mean ± standard deviation with individual data points. One-way ANOVAs and Tukey post hoc corrections to identify pairwise differences. Significant comparisons are denoted by solid bars. (* *p* < 0.05, ** *p* < 0.01, and *** *p* < 0.001).

The expression of matrix remodeling-associated genes was altered following *Col5a1* knockdown. *Adamts5* and *Prg4* expressions were significantly higher in injured WT control tendons compared with TM5 I-*Col5a1*^−/−^ tendons at 3 weeks post-injury (Figure 2A,B). At 6 weeks post-injury, *Mmp2* expression was higher in TM5 I-*Col5a1*^−/−^ tendons compared with injured WT tendons (Figure 2C). Likewise, *Mmp3* expression was elevated in both TM5 I-*Col5a1*^+/−^ and TM5 I-*Col5a1*^−/−^ tendons at 6 weeks post-injury relative to WT tendons (Figure 2D). TM5 I-*Col5a1*^−/−^ tendons exhibited higher *Aspn* expression compared to WT and TM21 I-*Col5a1*^−/−^ tendons at 6 weeks post-injury (Figure 2E).

#### 2.1.2. Fibrillogenesis Associated Genes

**Figure 3:**
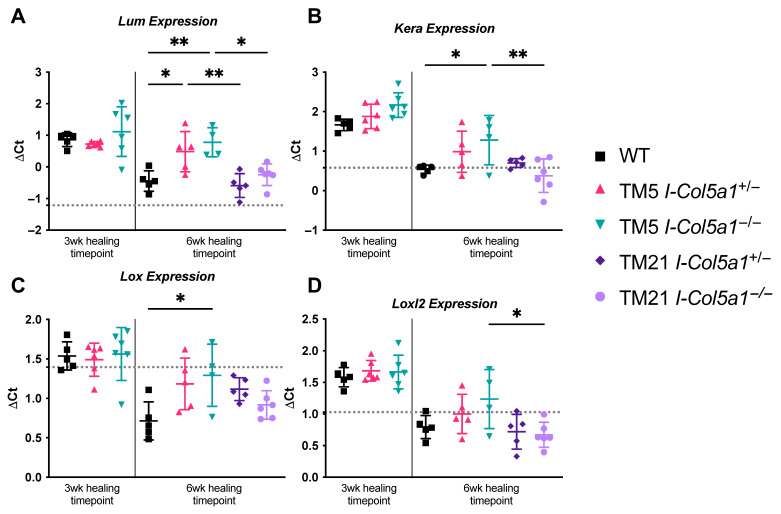
After 6 weeks of healing, fibrillogenesis-associated genes (**A**) *Lum*, (**B**) *Kera*, and (**C**) *Lox* expression in TM5_I-*Col5a1*^−/−^ tendons and (**A**) *Lum* expression in TM5_I-*Col5a1*^+/−^ tendons were upregulated relative to injured WT controls. (**A**) *Lum*, (**B**) *Kera* and (**D**) *Loxl2* expressions were also markedly reduced in *Col5a1* null tendons knocked down at 21 days post-injury (TM21_I-*Col5a1*^−/−^) relative to their counterparts that were induced at 5 days post-injury (TM5_I-*Col5a1*^−/−^). (**A**) *Lum* expression was reduced in the TM5_I-*Col5a1*^+/−^ tendons compared to TM21_I-*Col5a1*^+/−^. Dotted lines denote gene expression in uninjured WT tendons. Data is displayed as mean ± standard deviation with individual data points. One-way ANOVAs and Tukey post hoc corrections to identify pairwise differences. Significant comparisons are denoted by solid bars. (* *p* < 0.05, ** *p* < 0.01).

Genes associated with fibrillogenesis and proteoglycan regulation were significantly altered in response to collagen V knockdown. Lumican (*Lum*) expression was higher in TM5 I-*Col5a1*^+/−^ and TM5 I-*Col5a1*^−/−^ tendons at 6 weeks post-injury compared with WT tendons (Figure 3A). Across induction timepoints, TM5 I-*Col5a1*^+/−^ and TM5 I-*Col5a1*^−/−^ tendons had greater *Lum* expression compared to their TM21 counterparts, TM21 I-*Col5a1*^+/−^ and TM21 I-*Col5a1*^−/−^ (Figure 3A). Keratocan (*Kera*) expression was also increased following early *Col5a1* knockdown. TM5 I-*Col5a1*^−/−^ tendons at 6 weeks post-injury exhibited higher *Kera* expression relative to WT tendons at the same time point, as well as compared with TM21 I-*Col5a1*^−/−^ tendons (Figure 3B). At the 6-week healing time point, TM5 I-*Col5a1*^−/−^ tendons exhibited higher *Lox* (Figure 3C) and *Loxl2* (Figure 3D) expressions than WT and TM21 I-*Col5a1*^−/−^ tendons, respectively.

#### 2.1.3. Matrix Crosslinking, Tenogenic, and Injury-Responsive Markers

**Figure 4:**
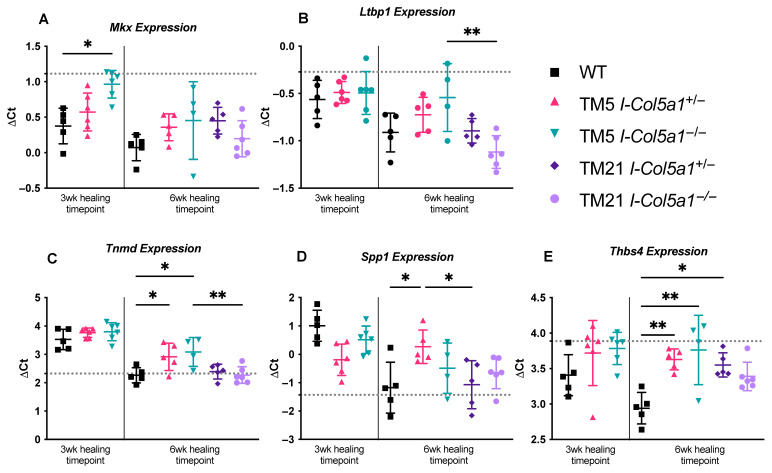
Expression of tendon gene marker (**A**) *Mkx* was increased in the TM5_I-*Col5a1*^−/−^ tendons induced at 5 days post-injury compared to the injured WT controls after 3 weeks of healing. (**B**) *Ltbp1* and (**C**) *Tnmd* expressions were reduced in tendons knocked down at 21 days post-injury (TM21_I-*Col5a1*^−/−^) relative to their TM5_I-*Col5a1*^−/−^ counterparts after 6 weeks of healing. Also, after 6 weeks of healing, (**C**) *Tnmd*, (**D**) *Spp1*, and (**E**) *Thbs4* expressions were significantly increased in tendons when *Col5a1* was knocked down at 5 days post-injury (TM5_I-*Col5a1*^−/−^ and TM5_I-*Col5a1*^+/−^) and 21 days post-injury (TM21_I-*Col5a1*^+/−^) relative to injured WT controls. (**D**) *Spp1* expression was also elevated in TM5_I-*Col5a1*^+/−^ tendons compared to TM21_I-*Col1a1*^+/−^ tendons. Dotted lines denote gene expression in uninjured WT tendons. Data is displayed as mean ± standard deviation with individual data points. One-way ANOVAs and Tukey post hoc corrections to identify pairwise differences. Significant comparisons are denoted by solid bars. (* *p* < 0.05, and ** *p* < 0.005).

The expression of tenogenic markers was also affected by *Col5a1* knockdown. Mohawk (*Mkx*) expression was higher in TM5 I-*Col5a1*^−/−^ tendons at 3 weeks post-injury compared with WT tendons at the same time point (Figure 4A). Similarly, the expression of latent TGF-β binding protein 1 (*Ltbp1*) was higher in TM5 I-*Col5a1*^−/−^ tendons compared to TM21 I-*Col5a1*^−/−^ tendons after 6 weeks of healing (Figure 4B). Tendomodulin (*Tnmd*), thrombospondin (*Thbs4*), and secreted phosphoprotein (*Spp1*) expressions were increased in inducible genotypes at later healing stages (Figure 4C–E). At 6 weeks post-injury, WT tendons exhibited lower *Tnmd* expression relative to TM21 I-*Col5a1*^+/−^ and TM21 I-*Col5a1*^−/−^ tendons, and TM21 I-*Col5a1*^−/−^ tendons had lower *Tnmd* expression than TM5 I-*Col5a1*^−/−^ tendons (Figure 4C). TM5 I-*Col5a1*^+/−^ tendons exhibited higher *Spp1* expression, commonly known as osteopontin, compared to both WT tendons and TM21 I-*Col5a1*^+/−^ tendons at 6 weeks post-injury (Figure 4D). *Thbs4* expression was higher in TM5 I-*Col5a1*^+/−^, TM5 I-*Col5a1*^−/−^, and TM21 I-*Col5a1*^+/−^ tendons compared to injured WT controls at the later healing stage (Figure 4E).

### 2.2. Histology

**Figure 5:**
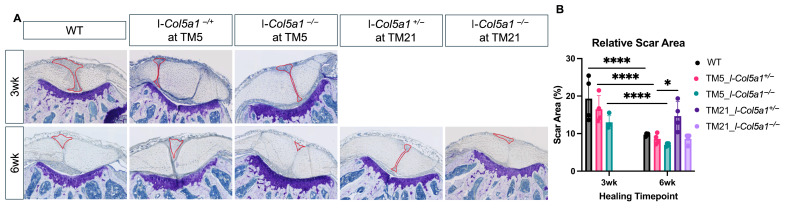
Injured tendon regions (*n* = 6/group) were stained with (**A**) toluidine blue to assess scar area in healing tendons at 3 weeks and 6 weeks following injury. (**B**) Quantification of relative scar area revealed that the scar area was reduced from 3 to 6 weeks of healing in all genotypes. The measured scar area is denoted by the red outline. Heterozygous tendons induced at 21 days post-injury (TM21_I-*Col5a1*^+/−^) had significantly larger scar area relative to their counterparts induced at 5 days post-injury (TM5_I-*Col5a1*^+/−^) after 6 weeks of healing. Data is displayed as mean ± standard deviation with individual data points. One-way ANOVAs and Tukey post hoc corrections to identify pairwise differences. Significant comparisons are denoted by solid bars. (* *p* < 0.05, and **** *p* < 0.0001).

We evaluated the effects of *Col5a1* knockdown at TM5 and TM21 post-injury on tendon scar formation by quantifying relative scar area within healing tendons after 3 and 6 weeks of healing. Injured tendon regions were identified in transverse tissue sections stained with toluidine blue (Figure 5A). As expected, the relative scar area significantly decreased in WT and both TM5 genotypes, TM5 I-*Col5a1*^+/−^ and TM5 I-*Col5a1*^+/−^, between 3 and 6 weeks of healing. No differences in relative scar area were observed between genotypes at 3 weeks post-injury. However, after 6 weeks of healing, TM21 I-*Col5a1*^+/−^ tendons exhibited a significantly greater relative scar area relative to TM5 I-*Col5a1*^+/−^ tendons (Figure 5B).

### 2.3. Fibril Diameter and Morphology

**Figure 6:**
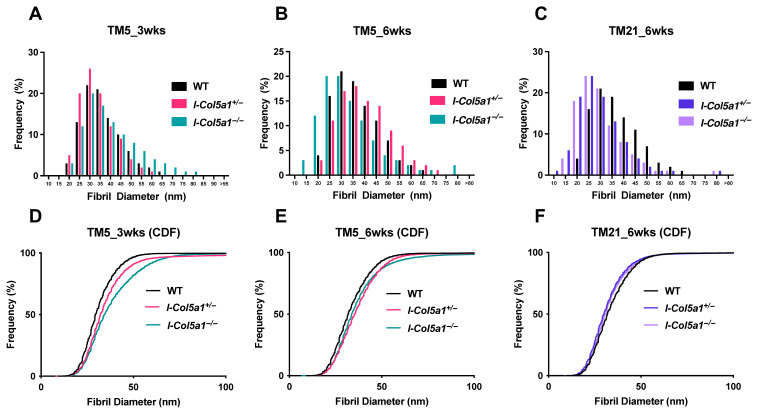
Injured wild-type (WT) fibril diameters are smaller and more unimodal relative to heterozygous (I-*Col5a1*^+/−^) and null (I-*Col5a1*^−/−^) knockdown tendons (**A**–**C**). Furthermore, at 3 and 6 weeks post-injury, TM5_I-*Col5a1*^−/−^ tendons had larger fibril diameters relative to TM5_I-*Col5a1*^+/−^. In total, 8000 to 10,000 fibrils were analyzed per genotype per time point. Cumulative distribution frequencies (**C**,**D**,**F**) also demonstrated these changes (**C**–**F**). Fibril diameter distributions were plotted in 5 nm bins with frequencies expressed as percentages; fibrils larger than 95 nm were assigned to a single “95+” bin. Data is displayed as averages.

Assessing collagen fibril diameter and morphology following injury provides insight into the role of collagen V in collagen fibrillogenesis. Healthy, uninjured tendons exhibit a characteristic bimodal distribution of collagen fibril diameters. As expected, injured WT tendons displayed a shift toward smaller fibrils and a unimodal distribution at both 3 and 6 weeks post-injury (Figure 6). At both 3 and 6 weeks post-injury, fibrils in TM5 I-*Col5a1*^+/−^ and I-*Col5a1*^−/−^ tendons exhibited larger diameters than those in WT tendons (Figure 6A,B,D,E). Quantitative fibril diameter analysis further demonstrated an overall rightward shift in the fibril diameter distribution, indicative of larger fibrils, in TM5 I-*Col5a1*^−/−^ tendons at 3 weeks post-injury. Notably, these distinct distributional shifts were not observed in TM21 tendons at 6 weeks post-injury. Within the TM5 group, fibril diameters were consistently greater in I-*Col5a1*^−/−^ tendons compared with I-*Col5a1*^+/−^ tendons at both healing time points, supporting an allele dose-dependent effect of collagen V knockdown.

Differences in fibril diameters among WT, I-*Col5a1*^−/−^, and I-*Col5a1*^+/−^ tendons were most pronounced at TM5 3 weeks post-injury (Figure 6D). This group also exhibited increased fibril diameter heterogeneity, with a substantially larger interquartile range (Q3–Q1 = 18.09) in TM5 I-*Col5a1*^−/−^ tendons compared with all other groups, which displayed interquartile ranges between 11.43 and 15.19 (Table 2). In contrast, fibril diameters were more similar across genotypes at later healing stages, with the least divergence observed in the TM21 group at 6 weeks post-injury (Figure 6F). In this group, WT tendons exhibited larger fibril diameters than both inducible genotypes, with I-*Col5a1*^+/−^ tendons displaying larger fibrils than I-*Col5a1*^−/−^ tendons (Figure 6C,F). Consistent with this pattern, TM21 tendons had lower mean and median fibril diameters in both inducible genotypes, suggesting impaired fibril growth when collagen V knockdown occurs during later stages of tendon healing.

**Figure 7:**
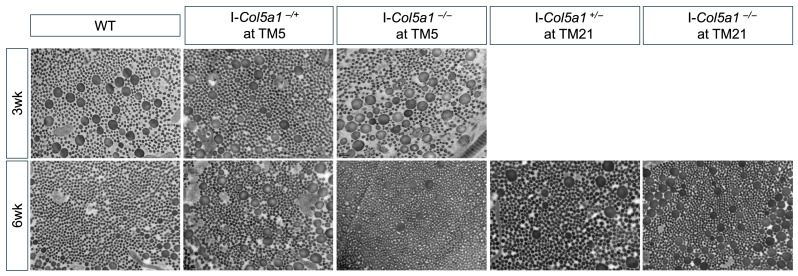
Transmission electron microscopy (TEM) visualized cross-sections of injured tendons, allowing for fibril diameters to be quantified. Scale bars denote 200 nm.

In addition to changes in fibril size, *Col5a1* deficiency altered fibril morphology. TM5 *Col5a1*^−/−^ tendons at 3 weeks post-injury exhibited the most pronounced morphological changes, characterized by a more irregular, punctuated fibril profile compared with the smoother, more rounded fibrils observed in the WT tendons (Figure 7). A similar, though less pronounced, alteration in fibril morphology was observed in TM5 I-*Col5a1*^+/−^ tendons at 6 weeks post-injury. Furthermore, transmission electron microscopy (TEM) imaging revealed a higher prevalence of smaller-diameter fibrils in TM21 I-*Col5a1*^−/−^ tendons.

### 2.4. Quasi-Statics Mechanical Properties

**Figure 8:**
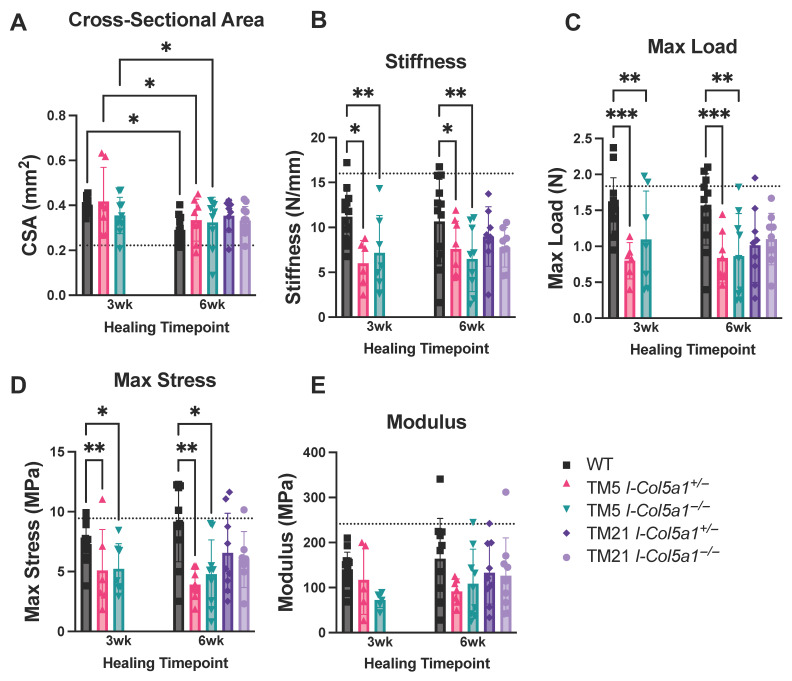
Cross-sectional area (**A**) decreased over time in injured wild-type (WT) controls and in heterozygous and null groups knocked down 5 days post-injury (TM5_I-*Col5a1*^+/−^ and TM5_I-*Col5a1*^−/−^, respectively). (**B**) Stiffness, (**C**) max load, and (**D**) max stress were significantly decreased in TM5_I-*Col5a1*^+/−^ and TM5_I-*Col5a1*^−/−^ tendons relative to injured WT controls after 3 and 6 weeks of healing. There were no significant differences in (**E**) modulus between healing time points and genotypes. Dotted lines denote uninjured WT tendons. Data is displayed as mean ± standard deviation with individual data points. One-way ANOVAs and Tukey post hoc corrections to identify pairwise differences. Significant comparisons are denoted by solid bars. (* *p* < 0.05, ** *p* < 0.005, and *** *p* < 0.001).

Quasi-static and viscoelastic mechanical testing was performed to evaluate the effects of *Col5a1* knockdown timing and allele dosage on tendon mechanical properties throughout healing. Tendon cross-sectional area (CSA) decreased over time in all groups, consistent with expected tissue remodeling during the healing process (Figure 8A). Structural mechanical properties were altered in tendons with early collagen V knockdown. At both 3 and 6 weeks post-injury, TM5 I-*Col5a1*^+/−^ and I-*Col5a1*^−/−^ tendons exhibited significantly reduced stiffness compared with WT tendons (Figure 8B). In contrast, no statistically significant differences in stiffness were observed between WT tendons and TM21 tendons at 6 weeks post-injury, regardless of genotype. Consistent with these findings, maximum load was reduced in TM5 I-*Col5a1*^+/−^ and I-*Col5a1*^−/−^ tendons at both 3 and 6 weeks compared to injured WT controls (Figure 8C). No significant differences in maximum load were observed between injured WT and TM21 control tendons at 6 weeks post-injury. Similar to the changes in structural properties, material properties were also altered with the loss of *Col5a1*. Maximum stress was reduced in TM5 I-*Col5a1*^+/−^ and I-*Col5a1*^−/−^ tendons at 3 and 6 weeks post-injury relative to injured WT control tendons (Figure 8D). Similar to stiffness and maximum load, no significant differences in maximum stress were observed between injured WT and TM21 tendons at 6 weeks post-injury. Tendon modulus did not differ significantly among genotypes or induction time points (Figure 8E).

**Figure 9:**
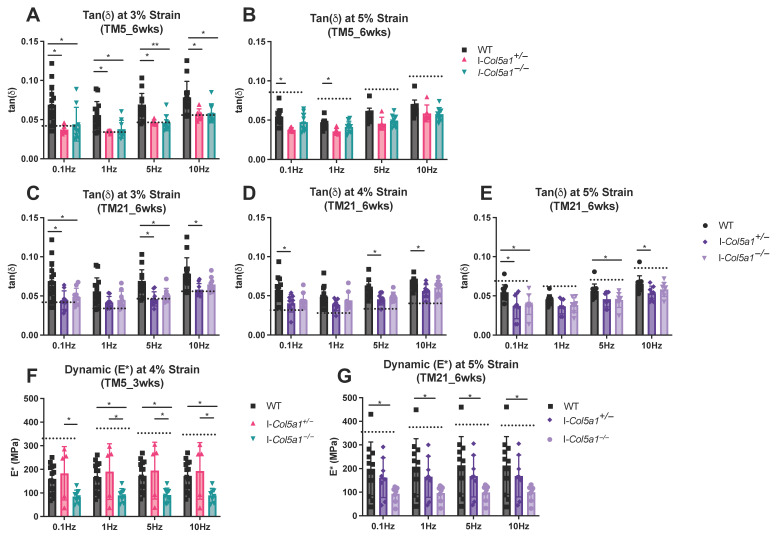
Dynamic mechanical analysis reveals strain-, frequency-, and time-dependent effects of collagen V depletion on tendon viscoelastic properties. At 6 weeks post-injury, tan δ differed between injured WT control (WT_6wks) and knockdown tendons across multiple strains and frequencies in both TM5 and TM21 groups (**A**–**E**), with more pronounced differences in TM5 (**A**). Dynamic modulus (E*) differences were limited, with reduced modulus in (**F**) TM5 tendons at 3 weeks (4% strain), indicating an allele dose-dependent effect, and later reductions in (**G**) TM21 tendons at 6 weeks (5% strain) compared to the healing time-matched injured WT control tendons. Data is displayed as mean ± standard deviation with individual data points. One-way ANOVAs and Tukey post hoc corrections were run to assess statistical differences. Bars denote statistical differences between groups. Dotted lines denote uninjured WT tendons. (* *p* < 0.05, ** *p* < 0.01).

Dynamic mechanical analysis revealed strain-, frequency-, and time-dependent effects of collagen V knockdown on tendon viscoelastic behavior. Differences in tan δ were only observed at 6 weeks post-injury. At 3% strain, tan δ of injured WT control tendons (WT_6wks) differed from that of TM5 I-*Col5a1*^+/−^ and I-*Col5a1*^−/−^ tendons across frequencies from 0.1 to 10 Hz (Figure 9A), and the injured WT tendons (WT_6wks) differed from I-*Col5a1*^+/−^ tendons at 0.1, 5, and 10 Hz, and from I-*Col5a1*^−/−^ tendons at 0.1 and 5 Hz at 6 weeks post-injury (Figure 9C). At 4% strain, tan δ of the injured WT control tendons differed from that of I-*Col5a1*^+/−^ tendons at 0.1, 5, and 10 Hz at 6 weeks post-injury (Figure 9D). At 5% strain, Tan δ differed between injured WT controls (WT_6wks) and TM5 I-*Col5a1*^+/−^ tendons over 0.1 and 1 Hz (Figure 9B), and injured WT control tendons (WT_6wks) differed from I-*Col5a1*^+/−^ tendons at 0.1 and 10 Hz, and from I-*Col5a1*^−/−^ tendons at 0.1 and 5 Hz at the 6-week healing time point (Figure 9E).

At 4% strain, dynamic modulus differed in the TM5 group at 3 weeks post-injury (Figure 9F). Specifically, injured WT tendons (WT_3wks) exhibited higher dynamic modulus compared with I-*Col5a1*^−/−^ tendons across frequencies from 1 to 10 Hz. In addition, both I-*Col5a1*^+/−^ and I-*Col5a1*^−/−^ tendons differed from injured WT (WT_3wks) across a broader frequency range (0.1–10 Hz), indicating an allele dose-dependent effect of collagen V knockdown at this early healing stage (Figure 9F). No differences in dynamic modulus were observed at 4% strain in TM5 or TM21 tendons at 6 weeks post-injury. At 5% strain, differences in dynamic modulus were observed only in the TM21 group at 6 weeks post-injury, where injured WT control tendons (WT_6wks) exhibited higher dynamic modulus than I-*Col5a1*^−/−^ tendons across frequencies from 0.1 to 10 Hz (Figure 9G).

## 3. Discussion

Collagen V is a quantitatively minor but functionally essential fibrillar collagen that regulates type I collagen fibril nucleation and lateral expansion [3,4]. Although its structural gatekeeper role during development is well established, its temporally restricted function during adult tendon repair has remained unclear. Using an inducible *Col5a1* knockdown model, we demonstrate that temporal *Col5a1* knockdown during either the late inflammatory phase (~5 days post-injury; TM5) or the early remodeling phase (~21 days post-injury; TM21) produces dose- and timing-dependent alterations in fibril architecture, transcriptional regulation, and mechanical behavior. Notably, early knockdown results in marked fibril dysregulation and diminished failure properties while largely preserving sub-failure mechanics, revealing a temporally sensitive requirement for collagen V during reparative matrix assembly.

A central finding is that collagen V function during tendon healing is temporally restricted. Early knockdown (TM5) produced a rightward shift in fibril diameter distribution, increased diameter heterogeneity, irregular morphology, and reduced stiffness, maximum load, and maximum stress. In contrast, delayed knockdown (TM21), imposed after initial establishment of the fibril matrix, had modest effects on quasi-static structural properties but selectively altered viscoelastic behavior and tenogenic gene expression at later stages. These data indicate that collagen V is most critical during early fibril nucleation and organization, whereas its influence diminishes once fibril scaffolds are established.

A striking feature of TM5 knockdown was a non-linear, allele dose-dependent response in fibril organization and mechanics. Heterozygous knockdown (TM5 I-*Col5a1*^+/−^) produced a modest increase in fibril diameter throughout healing relative to homozygous knockdown tendons (TM5 I-*Col5a1*^−/−^). This pattern suggests that partial reduction transiently permits greater lateral fibril growth without destabilizing fibril architecture. This phenotype likely reflects coordinated dysregulation of fibrillogenesis-associated regulators. Small leucine-rich proteoglycans (SLRPs), including lumican and keratocan, were significantly upregulated in TM5 tendons, particularly in homozygous knockdown animals. These molecules regulate fibril assembly, lateral growth, and interfibrillar spacing [13]. Lumican specifically modulates early fibril formation and organization [13] and SLRP deficiency alters fibril morphology and tendon mechanics [14]. Asporin, which binds collagen and competes with other SLRPs, further implicates disruption of the proteoglycan–collagen regulatory network [15,16]. Complementary evidence from collagen XI studies demonstrates that multiple regulatory collagens coordinate fibril diameter and assembly [16]. Together, these findings suggest that moderate collagen V reduction may transiently preserve a more optimal fibril distribution, whereas excessive loss disrupts regulatory balance and compromises matrix organization.

Early collagen V knockdown may have also altered matrix remodeling pathways in a biphasic manner. For example, a reduction in *Adamts5* and *Prg4* expressions at 3 weeks may influence downstream early matrix turnover and lubrication-associated remodeling. The *Prg4* gene encodes lubricin, a protein that contributes to tendon surface lubrication and remodeling processes [17], while ADAMTS proteases regulate ECM turnover during tendon repair [18]. By 6 weeks, however, TM5 tendons exhibited elevated *Mmp2* and *Mmp3* expressions, suggesting sustained or dysregulated proteolytic activity [19,20]. This shift from suppressed early turnover to later protease upregulation could suggest that initial fibrillogenesis defects trigger compensatory remodeling cascades that persist into later healing phases. Likewise, concomitant increases in *Lox* and *Loxl2* expressions may lead to downstream alterations in crosslinking activity, as lysyl oxidase family members mediate collagen crosslink formation and tensile strength development [4,21,22]. However, despite elevated crosslinking-associated gene expression, structural properties remained diminished. This disconnect supports a hierarchical model of matrix assembly in which proper fibril nucleation precedes and conditions effective crosslink formation [4]. These findings support the notion that once early nanostructural organization is disrupted, subsequent enzymatic stabilization cannot fully restore mechanical integrity.

Altered expression of latent transforming growth factor beta-1 (*Ltbp1*) and asporin (*Aspn*) suggests that collagen V knockdown may influence TGF-β-related signaling within the healing matrix. LTBP1 anchors latent TGF-β complexes and regulates their activation [23,24], while asporin binds TGF-β and modulates downstream signaling [25,26,27,28]. Thus, changes in *Ltpb1* and *Aspn* expressions in the knockdown tendon may reflect alterations in pathways associated with growth factor sequestration and matrix signaling during healing. Increased expression of periostin (*Postn*) in homozygous knockdown tendons may also be consistent with changes in mechanotransductive feedback mechanisms [29,30], as periostin expression is responsive to ECM organization and mechanical cues. Together, these transcriptional changes suggest that collagen V knockdown may impact signaling pathways linked to matrix remodeling and cellular responses to the extracellular environment.

Tenogenic markers displayed timing-specific responses. Elevated Mohawk (*Mkx*) expression in the TM5 null *Col5a1* knockdown (TM5 I-*Col5a1*^−/−^) tendons at 3 weeks may represent an early transcriptional response associated with restoration of the tendon phenotype following early structural disruption, consistent with the role of *Mkx* in fibril organization and tendon maturation [31,32,33,34]. Increased osteopontin (*Spp1*) in heterozygous TM5 tendons at 6 weeks is consistent with sustained injury-associated signaling, as *Spp1* is commonly linked to inflammation, fibrosis, and matrix remodeling [35]. In contrast, increased tenomodulin (*Tnmd*) expression in TM21 groups at later healing stages, coupled with preserved quasi-static mechanical properties, may reflect more advanced matrix maturation when collagen V knockdown occurs after the initial phases of fibril organization [36,37].

Collectively, these findings suggest that collagen V plays a critical role in fibrillogenesis during different stages of tendon healing. Collagen V is particularly important during the early phase of tendon healing. Early knockdown was associated with increased fibril heterogeneity and reduced structural mechanical properties, consistent with disruptions in early fibril organization. In contrast, delayed knockdown, applied after fibril scaffolds were established, resulted in more modest changes in quasi-static structural properties but was associated with alterations in viscoelastic behavior and gene expression. Together, these observations indicate that the timing of collagen V knockdown may differentially influence matrix organization and mechanical outcomes during repair, with a more pronounced impact when disrupted during early fibril formation.

These findings also align with previous work examining collagen V function across developmental, homeostatic, and reparative contexts. In mature tendon, constitutive *Col5a1* deletion disrupts fibril size and reduces failure strength [7]. In the context of tendon healing, we previously demonstrated that the magnitude of *Col5a1* suppression at the time of injury is associated with the severity of mechanical deficits [6]. Altogether, these studies support a broader framework in which collagen V dosage contributes to fibril assembly during development, maintenance of fibril nanostructure during homeostasis, and matrix organization during repair. The present study extends this framework by highlighting the potential importance of the timing of collagen V knockdown during the healing process.

## 4. Materials and Methods

### 4.1. Inducible Gene Ablation and Animal Surgery

Adult male C57BL/6J mice (p120, 20–30 weeks, 20–30 g) were used in all experiments. Three genotypes were studied: wild-type (WT, *n* = 45, Jackson Labs), homozygous inducible *Col5a1* knockdown (*I-Col5a1*^−/−^, *n* = 45), and heterozygous inducible *Col5a1* knockdown (*I-Col5a1*^+/−^, *n* = 45). All animals carried a ROSA26-CreERT2 allele, enabling tamoxifen-induced excision of floxed *Col5a1* alleles. Experiments were approved by the University of Pennsylvania Institutional Animal Care and Use Committee (IACUC, protocol no. 803267). At 120 days of age, mice received bilateral, partial-width, full-thickness patellar tendon injuries, performed under isoflurane anesthesia by a 0.75 mm biopsy punch centered on the midsubstance of the tendon. Following surgery, animals were monitored until recovery and then assigned to post-treatment groups. All mice were administered two intraperitoneal (IP) injections of tamoxifen (50 mg/kg) (TM) in corn oil. There were two separate injured WT mouse cohorts that served as Cre-negative controls at each healing time point, 3 and 6 weeks (denoted as injured WT control tendons). The injured WT control mice received TM injections on the day of and the day after surgery. Cre-mediated excision was performed on the I-*Col5a1*^−/−^ and I-*Col5a1*^+/−^ mice on two distinct occasions: at day 5 post-injury (TM5), corresponding to the late inflammatory phase, and at day 21 post-injury (TM21), corresponding to the remodeling phase. Injured WT and TM5 mice were sacrificed at 3 weeks and 6 weeks post-injury (WT_3wks, WT_6wks, TM5_3wks, and TM5_6wks) (*n* = 15/healing time point). TM21 mice were sacrificed at 6 weeks post-injury (TM21_6wks, *n* = 15). For all analyses, the injured WT control tendons from mice sacrificed at 6 weeks (WT_6wks) served as the shared comparator for both the TM5 and TM21 groups evaluated at the 6-week time point. A separate cohort of uninjured WT mice received 3 consecutive daily TM injections (100 mg/kg body weight) at 120 days old and were sacrificed 30 days later to provide a qualitative comparison to uninjured tendons (uninjured WT tendons, *n* = 15). Right patellar tendons were dissected; samples were either prepared for histology, transmission electron microscopy (TEM), or flash-frozen in liquid nitrogen, and stored at −80 °C for downstream gene expression. Whole bodies of all animals were stored at −20 °C to allow later retrieval for biomechanical testing.

### 4.2. Histology

For histological analysis (*n* = 6/group), the knee joint was isolated by cutting through the femur and tibia at the time of sacrifice. The knee was flexed to 90°, placed into a cassette, fixed in formalin, and processed using standard paraffin histological techniques. Samples were embedded in paraffin, and sections were cut at 7 μm thickness before staining with hematoxylin and eosin. Three sections were imaged per sample. A 400 × 500 μm region of interest within the tendon midsubstance of each image was selected for analysis. Cells were automatically segmented using a custom MATLAB script (R2026a). After manually selecting a region of interest to exclude non-tendinous tissue and empty space, the number of cells normalized to the area of the region of interest and the median nuclear aspect ratio of all cells within the region of interest were determined. Reported values for each region of interest were determined. Reported values for each biological replicate are averaged across the three sections for that replicate.

### 4.3. Gene Expression

Tendons (*n* = 4–6/genotype per timepoint) were thawed in RNAlater^®^ *ICE* (Thermo Scientific, Waltham, MA, USA), homogenized with plastic pestles in TRIzol^®^ reagent, and vortexed vigorously. Total RNA was isolated using the Direct-zol RNA MicroPrep kit (Zymo Research, Irvine, CA, USA) per the manufacturer’s instructions. cDNA synthesis was carried out with the High-Capacity cDNA Reverse Transcription Kit (Thermo Fisher Scientific, Waltham, MA, USA). For qPCR, cDNA was pre-amplified in a 15-cycle multiplex with TaqMan^®^ assays (Applied Biosystems, Foster City, CA, USA) for 96 target genes spanning categories of collagens, non-collagenous ECM proteins, matrix remodeling mediators, stress- and inflammation-responsive genes, cell-type markers, and housekeeping genes (Abl1 and Rps17). Pre-amplified cDNA was loaded onto a Fluidigm 96.96 Dynamic Array and processed on the BioMark™ system (Fluidigm, South San Francisco, CA, USA). Cycle threshold was measured for all genes. ΔCt values were calculated by subtracting the gene Ct values from the average housekeeper Ct value.

### 4.4. Transmission Electron Microscopy (TEM) Fibril Imaging

Patellar tendons (*n* = 4/genotype per timepoint) designated for ultrastructural analysis were prepared for transmission electron microscopy as described [38,39]. Briefly, tendons were fixed in 4% paraformaldehyde, 2.5% glutaraldehyde, 0.1 M sodium cacodylate, and 8 mM CaCl_2_ (pH 7.4). Samples were post-fixed in 1% osmium tetroxide for 1 h, dehydrated in graded ethanol, infiltrated with Epon 812 resin, and polymerized at 60 °C for 24 h. Ultrathin (∼90 nm) cross-sections were cut with an ultramicrotome and collected on Formvar-carbon-coated copper grids. Grids were stained with 2% uranyl acetate, followed by 1% lead citrate. Sections were imaged at 80 kV on a JEOL 1400 transmission electron microscope (Japan Electron Optics, Tokyo, Japan). From each tendon, 8–10 non-overlapping fields were photographed from the midsubstance. Images were randomized and anonymized before analysis. Collagen fibril diameters were measured along the minor axis of each fibril using BIOQUANT^®^ 2025 software (SAS, Chase City, VA, USA). A region of interest (ROI) was drawn in each image to include 8000 to 10,000 fibrils per sample (each genotype per time point), and measurements were pooled across ROIs. Diameter distributions were plotted in 5 nm bins with frequencies expressed as percentages; fibrils larger than 95 nm were assigned to a single “95+” bin.

### 4.5. Quasi-Static and Viscoelastic Mechanical Testing

Right patellar–tendon–tibia complexes (*n* = 14/genotype per timepoint) were thawed to room temperature, dissected, and stamped to produce a dog bone-shaped tendon specimen (∼10 mm length). The cross-sectional area (CSA) of each tendon was measured using a custom laser-based device. Specimens were mounted in a custom mechanical testing rig (Instron 5848, Norwood, MA, USA) with a 3-point load cell. Tibias were secured using polymethyl methacrylate in custom pots and the patellae were gripped with mechanical testing grips. Mechanical testing followed the protocol: 10 pre-conditioning cycles, stress-relaxation holds at 3%, 4%, and 5% strain, followed by 10 cycles of sinusoidal frequency sweeps (0.1, 1, 5, and 10 Hz) at each strain level. After a frequency sweep at 5% strain, the samples were returned to 0% strain and then subjected to a quasi-static ramp to failure at 0.1% strain per second. Samples that slipped at the grips, failed before completion of pre-conditioning, or displayed gross defects at the insertion site were excluded. CSA, stiffness, tangent modulus, maximum load, and maximum stress were calculated. Percent relaxation during each hold was calculated as the difference between the peak and final stress value divided by the peak stress. Dynamic modulus (E*) and phase shift (δ) were derived from the oscillatory data.

### 4.6. Statistical Analysis

Gene-expression, histological, and biomechanical data were first evaluated for normality using the Shapiro–Wilk test. For individual gene comparisons, a one-way ANOVA was applied to ΔCt values across genotypes at each healing time point, followed by Tukey post hoc corrections to identify pairwise differences. We acknowledge that this approach does not correct for multiple testing across the full gene panel. A formal multiple-comparison correction across genes was not applied because this analysis was designed as a targeted, hypothesis-driven evaluation of a predefined set of genes rather than an exploratory, high-throughput screen. Applying global corrections in this context may increase the risk of Type II error and mask biologically meaningful differences. Percent scar area and all mechanical end-points were examined with a two-way ANOVA that incorporated genotype and healing time as factors. Tukey adjustments were again used for multiple comparisons. Collagen fibril diameter distributions were assessed by Kolmogorov–Smirnov tests to determine morphological differences between groups within each time point, and diameter measurements were additionally binned into four quartiles per sample, with ANOVA and Tukey post hoc testing used to compare the relative frequency of each bin across genotypes. Statistical significance was set at *p* < 0.05. All findings were qualitatively compared to uninjured WT tendons to contextualize the impact of *Col5a1* knockdown and denoted as dotted lines on the graphs.

## 5. Conclusions

By prescribing *Col5a1* knockdown to defined healing windows, we demonstrate that collagen V suppression yields distinct structural and mechanical outcomes depending on timing. This temporal resolution underscores a narrow time window during which fibrillogenesis is particularly sensitive to perturbation, highlighting important therapeutic considerations for strategies aimed at modulating collagen V. These findings carry translational relevance for connective tissue disorders such as classical Ehlers–Danlos syndrome (cEDS), in which *COL5A1* haploinsufficiency results in abnormal fibril architecture and compromised tissue integrity [8,9,10,12]. The exaggerated remodeling and compensatory proteoglycan upregulation observed following early knockdown recapitulate key features of developmental *Col5a1* deficiency, suggesting that the timing of collagen V modulation critically influences scar quality and functional recovery.

More broadly, this study demonstrates that matrix composition during early healing stages determines long-term mechanical performance. Interventions designed to optimize early fibrillogenesis or stabilize initial matrix assembly may therefore prove more effective than therapies applied during later remodeling phases. By defining collagen V as a dose- and time-sensitive contributor to tendon repair, this study establishes a framework linking gene regulation, fibril nanostructure, and biomechanics across discrete stages of healing.

Future studies will incorporate protein-level analyses for key matrix constituents (e.g., collagen V, decorin, asporin, and collagen XI) to further resolve transcription–translation relationships. Additionally, as this work was conducted on male mice using a patellar tendon injury model, future investigations are needed to determine whether these findings extend to female mice or other tendon injury models. Addressing these questions will clarify whether transient collagen V modulation can be therapeutically harnessed to enhance tendon repair without compromising matrix integrity.

## Figures and Tables

**Table 1:** Summary of gene expression across genotypes, WT, *I-Col5a1*^+/−^, and *I-Col5a1*^−/−^ (columns), and healing time points at 3 and 6 weeks (rows). Only upregulated genes are presented and indicated by upward arrows. The TM21 *I-Col5a1*^−/−^ group is excluded, as no genes were significantly upregulated relative to the other groups.

	WT	TM5 I-*Col5a1*^+/−^	TM5 I-*Col5a1*^−/−^	TM21 I-*Col5a1*^+/−^
**3 weeks**	↑ Adamts5 (vs. TM5 I-Col5a1^−/−^) ↑ Prg4 (vs. TM5 I-Col5a1^−/−^)		↑ Mkx (vs. WT)	
**6 weeks**		↑ Ltbp1 (vs. TM21 I-Col5a1^−/−^) ↑ Tnmd (vs. WT) ↑ Spp1 (vs. WT & TM21 I-Col5a1^−/−^) ↑ Thbs4 (vs. WT) ↑ Mmp3 (vs. WT) ↑ Lum (vs. WT & TM21 I-Col5a1^+/−^)	↑ Tnmd (vs. WT & TM21 I-Col5a1^−/−^) ↑ Mmp2 (vs. WT) ↑ Mmp3 (vs. WT) ↑ Thbs4 (vs. WT) ↑ Aspn (vs. WT & TM21 I-Col5a1^−/−^) ↑ Lum (vs. WT & TM21 I-Col5a1^−/−^) ↑ Kera (vs. WT & TM21 I-Col5a1^−/−^) ↑ Lox (vs. WT) ↑ Loxl2 (vs. TM21 I-Col5a1^−/−^)	↑ Thbs4 (vs. WT)

**Table 2:** First quartile (Q_1_), median (Q_2_), third quartile (Q_3_), and interquartile ranges (Q_3_–Q_1_) for fibril diameters for injured wild-type (WT), heterozygous (I-*Col5a1*^+/−^), and null (I-*Col5a1*^−/−^) tendons across induction and healing time points.

	Genotype	Q_1_	Q_2_	Q_3_	Q_3_–Q_1_
**TM5_3wks**	WT	24.73	28.44	36.16	11.43
	I-*Col5a1*^+/−^	26.95	32.06	40.04	13.09
	I-*Col5a1*^−/−^	27.33	34.17	45.42	18.09
**TM5_6wks**	WT	26.1	31.81	40.05	13.95
	I-*Col5a1*^+/−^	28.32	35.4	43.46	15.14
	I-*Col5a1*^−/−^	27.33	33.66	42.52	15.19
**TM21_6wks**	WT	26.1	31.81	40.05	13.95
	I-*Col5a1*^+/−^	24.63	30.03	36.72	12.09
	I-*Col5a1*^−/−^	25.33	30.45	38.05	12.72

## Data Availability

The original contributions presented in this study are included in the article. Further inquiries can be directed to the corresponding author.

## Abbreviations

The following abbreviations are used in this manuscript:

ECM	Multidisciplinary Digital Publishing Institute
TM5	Tamoxifen-Induced at 5 Days Post-Injury
TM21	Tamoxifen-Induced at 21 Days Post-Injury
*Col5a1*	Collagen V Gene Expression
*Adamts5*	A Disintegrin and Metelloproteinase with Thrombospondin Motifs 5
*Prg4*	Proteoglycan 4
*Mmp3*	Matrix Metalloproteinase 3
*Mmp2*	Matrix Metalloproteinase 2
*Aspn*	Asporin
*Lum*	Lumican
*Kera*	Keratocan
*Lox*	Lysyl Oxidase
*Loxl2*	Lysyl Oxidase Like 2
*Mkx*	Mohawk
*Ltbp1*	Latent Transforming Growth Factor Beta Binding Protein 1
*Tnmd*	Tenomodulin
*Spp1*	Secreted Phosphoprotein 1
*Thbs4*	Thrombospondin 4
